# Dural arteriovenous fistula presenting with stroke-like symptoms and regional cerebral hyperperfusion: a case report

**DOI:** 10.1186/s12883-023-03229-z

**Published:** 2023-05-04

**Authors:** Hanfeng Chen, Ruili Wei, Ziqi Xu

**Affiliations:** Department of Neurology, The First Affiliated Hospital, College of Medicine, Zhejiang University, Hangzhou, China

**Keywords:** Dural arteriovenous fistula, Ischemic stroke, Misdiagnosis, Cerebral hyperperfusion

## Abstract

**Background:**

Non-hemorrhagic focal neurological deficit is one of the clinical manifestations of intracranial dural arteriovenous fistulas (DAVF). When symptoms appear suddenly, it is difficult to distinguish it from ischemic stroke in certain circumstances, which might easily lead to misdiagnosis. Here, we report a rare case of DAVF with sudden onset sensory aphasia mimicking hyperacute stroke but presented with unexpected regional hyperperfusion on the site corresponding to its symptoms.

**Case presentation:**

A 76-year-old male with histories of atrial fibrillation and hypertension was admitted to the emergency department due to sudden sensory aphasia. The diagnosis of ischemic stroke was made based on clinical experience after non-contrast CT excluding hemorrhage. As in the absence of clear contraindication, the patient received intravenous thrombolysis. On the cerebral CT perfusion, the left temporal lobe, where the sensory speech center is located, was manifested as regional hyperperfusion. Thrombolysis was subsequently halted, but scheduled cranial imaging indicated hemorrhagic transformation. According to the radiological hint from cranial MRI, the patient was suspected of having DAVF, which was finally confirmed by cerebral digital subtraction angiography.

**Conclusion:**

When DAVF is presented as sudden onset focal neurological deficit, cranial CT perfusion at an early stage might reveal an abnormal hyperperfusion pattern. Clinicians should be aware of the diagnostic possibility of DAVF in this situation and double-review the CT angiography image to reduce missed diagnoses.

## Background

Intracranial dural arteriovenous fistula (DAVF) is an unusual cerebral vascular malformation defined by an abnormal connection between branches of arteries and veins in the dura mater. The incidence of DAVF is reported to be approximately 0.15–0.29 per 100,000 persons per year and comprises 10-15% of intraparenchymal arteriovenous malformations [1–3]. As affected by the different locations of the fistula and multiple venous drainage patterns, the clinical manifestations of DAVF are widely varied, ranging from vague symptoms such as headache or tinnitus to disabling focal neurologic deficits or a life-threatening intracranial hemorrhage [3]. Among them, non-hemorrhagic focal neurological deficits are fairly uncommon. It is quite challenging to promptly diagnose a DAVF at an early stage with this nonspecific symptom as the initial manifestation, especially when the patient is admitted in emergencies. Herein, we report a case of intracranial DAVF presented with sudden onset of sensory aphasia, which was initially misdiagnosed as ischemic stroke and received intravenous thrombolysis. The corresponding cerebral area was manifested as regional hyperperfusion on the CT perfusion map, which was seldom reported before.

## Case Presentation

A 76-year-old man was admitted to our emergency department by ambulance at 19:37 because of his sudden speech disturbance for 40 min. Medical records revealed a previous diagnosis of hypertension for eight years and chronic atrial fibrillation for six years. He was on oral antihypertension drugs but did not receive anticoagulant treatment. His family history was negative for any neurological disorder, and he denies histories of tinnitus, head trauma, or cranial surgery. On neurological examination, the patient was alert but notable for fluent aphasia with paraphrastic errors and inability to name, read, or write. The cranial nerves were intact, and all modalities of sensation or muscle strength were normal. Physical examination revealed body temperature of 36.8 °C, heart rate of 84 beats per minute, respiration rate of 20 breaths per minute, and blood pressure of 158/76 mm Hg (1 mm Hg = 1.33 kPa).

**Fig. 1:**
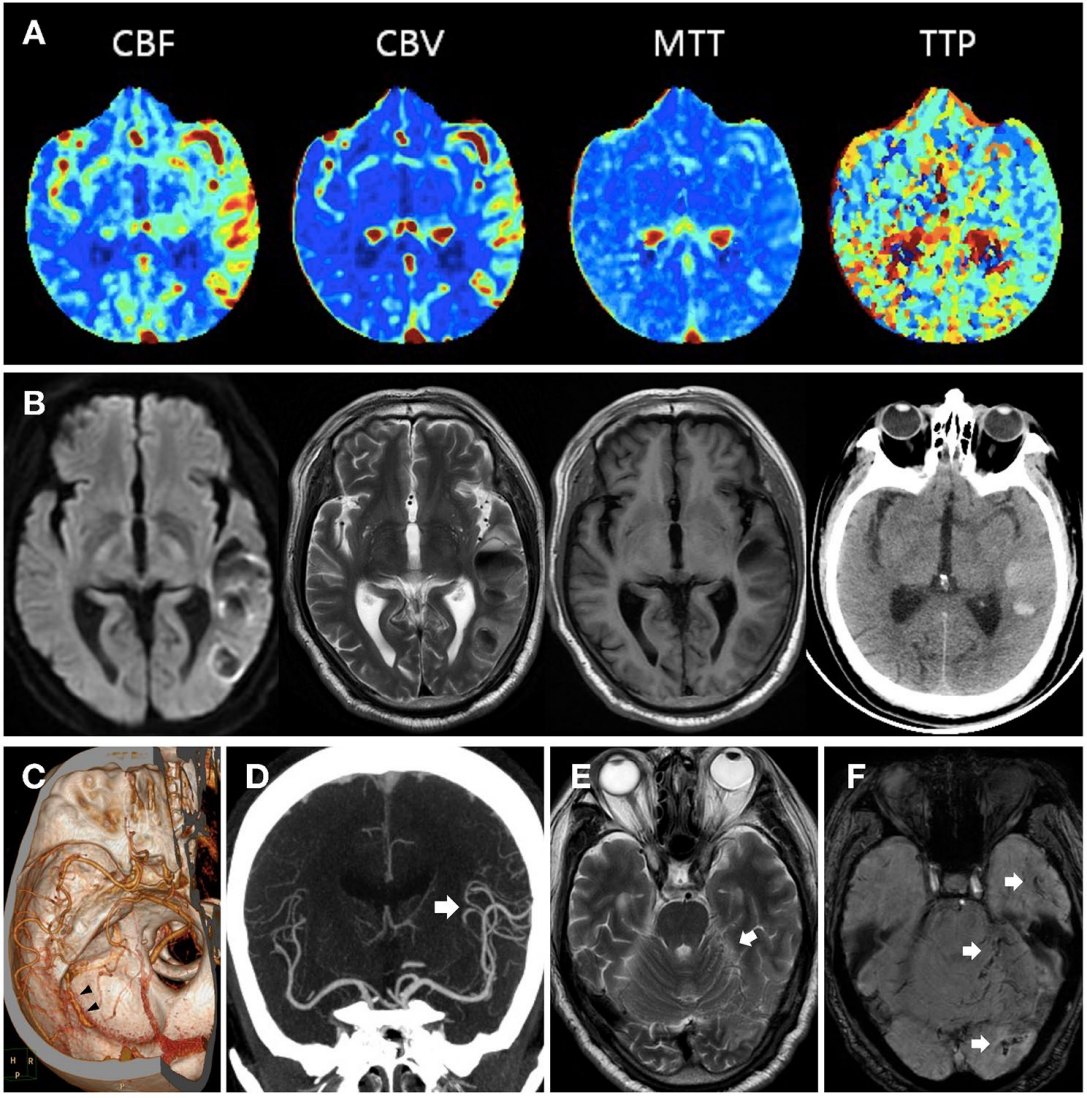
Cranial imaging appearance of the patients. (**A**) CT perfusion showed regional hyperperfusion in the left temporal lobe, with characteristics of a significant increase in cerebral blood flow (CBF), a mild increase in cerebral blood volume (CBV), and a prolonged mean transit time (MTT) and time to peak (TTP). (**B**) Cranial MRI and CT indicated hemorrhagic transformation in the left temporal lobe after intravenous thrombolysis. (**C**) Cranial CTA showed an early visualized left transverse sinus segment (black arrowhead), which was previously ignored and misinterpreted as venous contamination. (**D**) Hypervascularity in the left cerebral hemisphere (white arrow) was noted in cranial CTA, consistent with the regional hyperperfusion pattern. (**E**) Cranial MRI T2 sequence revealed tortuous abnormal vascular flow voids (white arrow) in the left temporo-occipital lobe and cerebellum. (**F**) Cranial susceptibility-weighted imaging showed clusters of engorgement veins, thought to represent collateral venous flow (white arrow)

Emergency cranial non-contrast CT was ordered, and the result showed neither intracranial hemorrhagic nor early ischemic changes. Extensive laboratory examinations were unremarkable, including complete blood count, glucose, electrolytes, coagulation studies, and hepatic and renal function tests. Given the acute onset of the clinical manifestation and a history of atrial fibrillation without anticoagulation, cerebrovascular embolism involving the sensory speech center in the dominant hemisphere’s temporal lobe was considered the most probable diagnosis. At 36 min from admission, the patient received intravenous thrombolysis on a standard dose of alteplase (0.9 mg/kg body weight), with the onset-to-needle time being 73 min. Before thrombolysis, the patient had a score of 6 on the National Institutes of Health Stroke Scale (NIHSS), and the modified Rankin scale (mRS) was 2. In order to determine whether there is intracranial large vessel occlusion and the necessity of bridge thrombectomy, multimodal CT imaging, including a head-and-neck CT angiography (CTA) and cerebral CT perfusion, was immediately scheduled after initiation of thrombolysis. The results indicated that the intracranial vessels were clearly visualized, with no large vessels or branches occluded. Regional hyperperfusion status in the left temporal lobe was recognized on the CT perfusion, with characteristics of an increase both in cerebral blood flow and cerebral blood volume (Fig. 1). As the hemodynamic change of hyperperfusion was sometimes compatible with seizure activity, a bedside electroencephalogram (EEG) was performed to eliminate the possibility of focal seizures. On examination, the patient was still symptomatic, but synchronized EEG revealed only intermittent polymorphic delta wave changes in the left temporal region without typical epileptiform discharge. Considering the clinical symptoms and signs could be explained by the involvement of the left temporal lobe, the patient was regarded as having cerebral infarction but experienced early vessel recanalization accompanied by post-recanalization hyperperfusion. Intravenous thrombolysis was halted 39 min into thrombolysis in fear of hemorrhagic transformation, with the actual dosage of alteplase being 65% of the standard. The patient’s vital signs were stable during the thrombolysis, with no new neurological signs or headaches occurring. The patient was transferred to the stroke unit ward for further monitoring.

Twelve hours after thrombolysis the following day, the patient’s speech disturbance was partially recovered, with the NIHSS score decreased to 3. And a cranial MRI was scheduled to localize the lesions. Unexpectedly, heterogeneous signal abnormalities in the left temporal lobe were presented, subsequently confirmed by the cranial CT being the intraparenchymal hemorrhage (Fig. 1). Surgical intervention was not warranted as the hematoma was localized with no significant occupying effect. Thus the patient was on continuous monitoring and symptomatic treatment. On the seventh day, cranial CT showed obvious absorption of the hemorrhage. The symptoms of aphasia were further improved, leaving only occasional spelling mistakes.

**Fig. 2:**
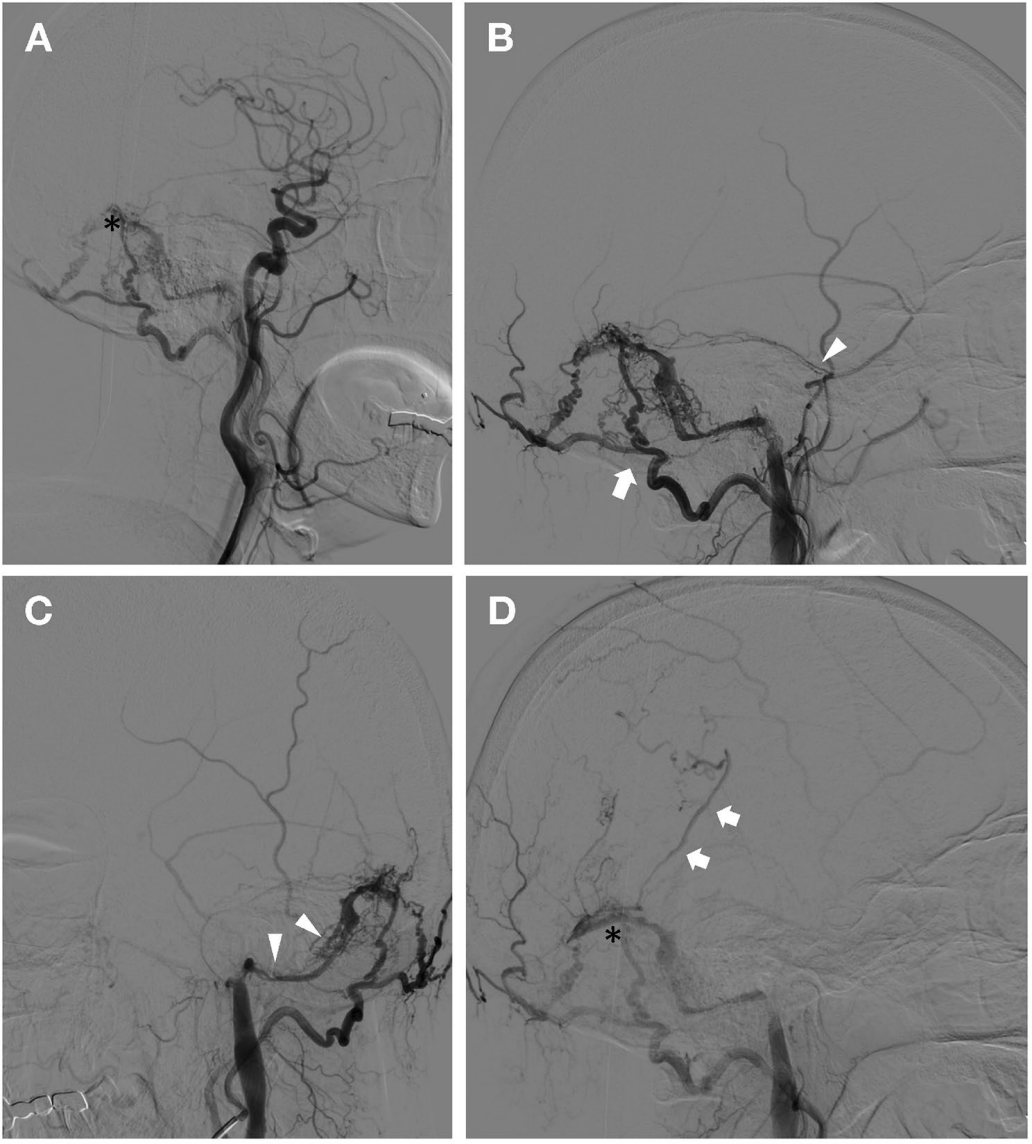
Cerebral digital subtraction angiography of the patients. (**A**) The Cognard type IIb dural arteriovenous fistula in the transverse sinus (black asterisk) is observed on digital subtraction images of a cerebral angiogram after left common carotid artery contrast injection. (**B**) The fistula was filled via shunts fed by the left occipital artery (white arrow) and branches of the middle meningeal artery (white arrowhead). (**C**) The downstream left sigmoid sinus (white arrowhead) appeared severe stenosis. (**D**) Retrograde venous drainage from the left transverse sinus (black asterisk) back into the vein of Labbe (white arrow) was observed during the late phase of cerebral angiogram

Cranial susceptibility-weighted imaging (SWI) was also performed during the hospitalization to evaluate the possible cerebral microbleeds burden as post-thrombolysis hemorrhage occurred. The results indicated no evidence of cerebral microbleed but conspicuous venous vasculature in the left temporo-occipital lobe and cerebellum (Fig. 1). When reevaluating the initial CT angiography imaging, early visualization of the left transverse sinus segment, previously described as venous contamination, had regained our attention (Fig. 1). Cerebral digital subtraction angiography (DSA) was ordered, which revealed a left transverse sinus and sigmoid sinus DAVF (TS-SS DAVF) supplied mainly by the ipsilateral occipital artery and branches of the middle meningeal artery. The increased shunt flow from the fistula and combined downstream stenosed sigmoid sinus caused venous hypertension and retrograde venous drainage directly into cortical veins (Fig. 2). The patient refused endovascular embolization and was free of new symptoms on a 3-month clinical revisit.

## Discussion and conclusions

Intracranial DAVF is a rare vascular condition where abnormal arteriovenous shunts are made within the dura mater. As DAVF tend to present later in life between the ages of 40 and 60, they are now thought to be acquired lesions [3]. Common predisposing factors reported include cerebral venous sinus thrombosis, head trauma, ear infection, and previous cranial surgery. Although the exact pathogenesis for the formation of DAVF is unclear, secondary venous hypertension due to venous thrombosis is thought to play a critical role [4]. Non-hemorrhagic focal neurological deficit is a rare clinical manifestation of DAVF, and its diagnosis error or delay may lead to poor prognosis or even make patients receive potentially harmful treatment. For the possible pathomechanism, vascular steal or postictal effect of seizure was postulated to explain the symptom [5]. Previous research focused on hemodynamic features in DAVF with focal neurologic deficits. The results indicated that reduced regional cerebral blood flow was correlated well with the site corresponding to the symptoms [6]. In this case, we demonstrate a patient with intracranial DAVF who presented with sudden onset of focal neurologic deficits mimicking ischemic stroke. On the emergency cerebral CT perfusion map, rather than reduced regional cerebral blood flow, the related left temporal lobe explaining the sensory aphasia was manifested as hyperperfusion. According to our knowledge, no similar case with direct evidence of cerebral hyperperfusion was ever reported. Regional cerebral hyperperfusion, sometimes called luxury perfusion syndrome, is a focal increase in cerebral blood flow accompanied by a reduction in cerebral oxygen extraction fraction. This phenomenon is occasionally seen in ischemic stroke patients with cerebral vessels recanalized by mechanical thrombectomy [7, 8]. And the mechanism is thought to be hemodynamic changes secondary to tissue metabolic acidosis [9]. Therefore, we initially mistakenly believed the hypervascularity in this reported case was explained by the early vessel recanalization after cerebral infarction. However, the final correct diagnosis of intracranial DAVF was made through clinical follow-up and cerebral imaging reevaluation.

In this case, the non-hemorrhagic focal neurological deficit with regional cerebral hyperperfusion, which differs from previously reported hypoperfusion, might reflect the adaptive changes in hemodynamics during an early stage in DAVF. With the presence of TS-SS DAVF, the arterial pressure would directly retrograde transmitted into the cerebral venous system, causing cortical venous hypertension and then impairing parenchymal venous drainage. When the metabolic wastes cannot be effectively cleared, a regional metabolic acidosis environment is created and thus stimulate the inflow artery to dilate, temporarily leading to regional cerebral hyperperfusion. However, this hypervascularity hemodynamics adaptive changes might only occur in the early stage of the disease. As cortical vein retrograde reflux and venous hypertension cannot be relieved, the cerebral blood flows would adaptively turn to supply the surrounding normal tissue, causing the consequent hypoperfusion of the affected area. Another possible explanation for this focal neurological deficit and regional cerebral hyperperfusion was correlated with epileptic seizure activity [10]. Due to cortical venous reflux and congestion, the adjacent cerebral parenchyma might experience vasogenic edema accompanied by blood-brain barrier destruction, thus leading to abnormal neuronal discharge. Although the synchronized bedside EEG showed only regional slowing waves without electrographic seizures, it still remains the possibility to judge the neurological symptoms of speech disorders as postictal status.

Because the mechanism of the focal neurological deficit caused by DAVF is non-thrombotic, intravenous thrombolysis was ineffective and instead increased the risk of hemorrhagic transformation. Case of intracranial DAVF misdiagnosed as ischemic stroke and experienced fatal intracranial hemorrhage after intravenous thrombolysis has also been reported before [11]. Our patient was also initially diagnosed with ischemic stroke and received intravenous thrombolysis without apparent contradiction. However, thrombolysis was immediately halted when the unexpected regional hyperperfusion was observed on the cerebral CT perfusion. It might partially explain why the hemorrhage was localized and did not cause serious consequences. Although no signal abnormality was found on cranial non-contrast CT, we assume that the anatomical basis of DAVF-induced non-hemorrhagic focal neurological deficits is cerebral parenchyma vasogenic edema caused by cortical venous hypertension/congestion. It is a pity we did not have the patient complete the cranial MRI before thrombolysis, and the subsequent hemorrhagic transformation masked the original lesion.

Cerebral DSA is the gold standard for diagnosing DAVF, but its invasive characteristics limit its extensive application. Although cranial MRI and conventional CT angiography are inferior in sensitivity in diagnosing DVAF to cerebral DSA, recognition of tiny radiological signs in the image by experienced physicians can help screen suspected patients promptly. Direct abnormal connection between arteries and veins and early visualized drainage veins are the main criteria for diagnosing DAVF on CTA. However, they were easily ignored in patients with low flow and concealed fistula. Due to venous hypertension and cortical venous reflux, venous engorgement would occur around the site of the fistula, which could manifest as abnormal vascular flow void on conventional T2 sequence. Furthermore, secondary ischemia urges the brain parenchyma to increase oxygen extraction from the vessel, which results in more desaturation of blood in the veins and together contributes to the prominence of the venous structure on SWI [12].

In conclusion, dural arteriovenous fistulas are heterogeneous vascular abnormalities with clinical symptoms and prognosis highly dependent on the fistula’s location and the venous drainage pattern. Venous hypertension and secondary arterial changes were considered as possible pathomechanisms. When the venous pressure is transmitted to a more restricted region, the behavioral syndrome will present as focal neurological deficits, which might be indistinguishable from that arising from an ischemic stroke. From the lesson learned from this case, when the emergency cerebral CT perfusion result does not meet the expected cerebral ischemic hypoperfusion pattern but instead indicates regional hyperperfusion, clinicians should include the possibility of DAVF as the differential diagnosis. Although some literature indicates hyperperfusion results on cerebral CT perfusion should not affect the decision of thrombolysis as an accompanied seizure can be the presenting feature of stroke [13], we strongly suggest carefully double-review the cranial CTA and reconsidering the risk of intravenous thrombolysis.

## Data Availability

The datasets used or analyzed during the current case report are available from the corresponding author on reasonable request.

## Abbreviations

DAVF: dural arteriovenous fistula
DSA: digital subtraction angiography
CTA: computed tomography angiography
CTP: computed tomography perfusion
SWI: susceptibility-weighted imaging
NIHSS: National Institutes of Health Stroke Scale
mRS: modified Rankin Scale
EEG: electroencephalogram
CBF: cerebral brain flow
CBV: cerebral brain volume
MTT: mean transmit time
TTP: time to peak
